# Exotic topological density waves in cold atomic Rydberg-dressed fermions

**DOI:** 10.1038/ncomms8137

**Published:** 2015-05-14

**Authors:** Xiaopeng Li, S Das Sarma

**Affiliations:** 1 Department of Physics, Condensed Matter Theory Center and Joint Quantum Institute, University of Maryland, College Park, Maryland 20742-4111, USA

## Abstract

Versatile controllability of interactions in ultracold atomic and molecular gases has now reached an era where quantum correlations and unconventional many-body phases can be studied with no corresponding analogues in solid-state systems. Recent experiments in Rydberg atomic gases have achieved exquisite control over non-local interactions, allowing novel quantum phases unreachable with the usual local interactions in atomic systems. Here we study Rydberg-dressed atomic fermions in a three-dimensional optical lattice predicting the existence of hitherto unheard-of exotic mixed topological density wave phases. By varying the spatial range of the non-local interaction, we find various chiral density waves with spontaneous time-reversal symmetry breaking, whose quasiparticles form three-dimensional quantum Hall and Weyl semimetal states. Remarkably, certain density waves even exhibit mixed topologies beyond the existing topological classification. Our results suggest gapless fermionic states could exhibit far richer topology than previously expected.

## Abstract (web summary)

Rydberg atomic gases have proven a fertile playground for exploring exotic phases of condensed matter systems. Here, Li and Das Sarma study Rydberg-dressed atomic fermions in a 3D optical lattice and find a series of mixed topological density wave phases having no analogue in conventional materials.

## Graphical abstract

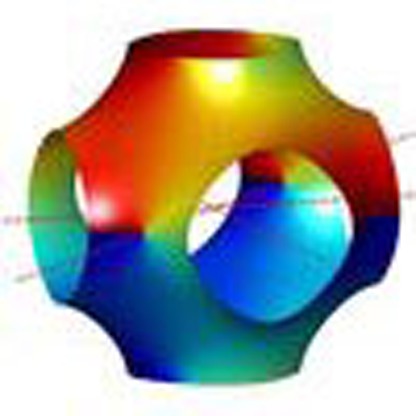

Topological quantum states of matter have attracted considerable theoretical and experimental attention in condensed matter research in the last decade, aiming at robust physical properties protected (often due to some underlying symmetry) against variations in microscopic details. For non-interacting fermions, topology of gapped phases has been classified for arbitrary dimensions^1^,^2^. Examples are Z class Chern insulators in two dimensions manifesting quantum anomalous Hall effect^3^,^4^, and time-reversal invariant Z_2_ topological insulators in three dimensions with robust magnetoelectric response^5^,^6^,^7^,^8^. For classification of gapless systems, no unified theoretical framework has emerged yet; nonetheless, recent studies on semimetals suggest one theoretical route by characterizing topological properties of band touching points, such as flux monopoles^9^,^10^,^11^,^12^,^13^.

In the context of ultracold atoms, recent experimental developments have realized quantum control of Rydberg excitations^14^,^15^,^16^,^17^,^18^,^19^,^20^, where strong van der Waals interactions lead to long-range interacting quantum systems. This allows novel collective quantum phenomena to be explored qualitatively beyond what are possible with the usual short-range interactions in atomic samples. In particular, the experimental scheme of off-resonant Rydberg dressing^19^,^21^,^22^ permits long life-time systems with controllable interaction strength and non-locality, which would promise exotic many-body phases such as supersolid^21^,^22^,^23^, quantum spin ice^24^ and topological Mott states^25^.

Here we provide one fascinating possibility in a Rydberg-dressed atomic Fermi gas beyond the present theoretical paradigm, with mixed gapped and gapless topologies emergent in a three-dimensional (3D) bond density wave state. We demonstrate the existence of such an exotic topological density wave via renormalization group (RG) and self-consistent calculations. Although our predicted density waves have some superficial similarity with various density wave phases being explored in the context of high-T_c_ cuprate superconductors^26^,^27^,^28^,^29^,^30^, the collective phases we theoretically predict here have no existing analogues. We also propose an experimental scheme to extract the topological properties based on time-of-flight signals.

## Results

### Density wave instability in Rydberg-dressed fermions

We consider a Rydberg-dressed atomic Fermi gas trapped in a 3D cubic optical lattice. This Rydberg atomic system has density–density interactions described by a non-local potential (see Methods for experimental realization), *V*(**r**)=*V*_6_/[1+(|**r**|/*r*_c_)^6^], where *V*_6_ describes the interaction strength and *r*_c_ the interaction range determined by the Condon radius in Rydberg dressing^19^,^21^,^22^,^24^,^25^. Considering a deep lattice, the kinetic motion of atoms arises mainly from quantum tunnelling between nearest neighbouring sites providing a unique kinetic energy scale, *t*. We will focus on a lattice with octahedral O_h_ symmetry. The model Hamiltonian to describe this system is *H*=*H*_0_+*H*_int_ with


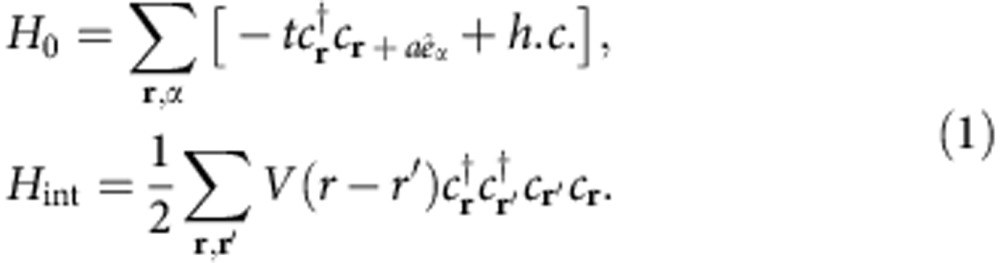


Here *a* is the lattice spacing, *α* index the three directions *x*, *y* and *z* and *c*_**r**_ the fermionic annihilation operator positioned at **r**. For fermionic atoms considered here, *V*(0) is set to be 0. The intrinsic properties of this system will depend on only two dimensionless parameters—*V*_6_/*t* and *r*_c_/*a*, representing the strength of interaction and its non-locality, respectively.

At half filling, namely one atom per two lattice sites, the Fermi surface is perfectly nested in the deep lattice limit, because the single-particle energy dispersion satisfies 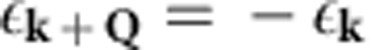, with **Q**=(*π*/*a*, *π*/*a*, *π*/*a*) (see Methods). The susceptibility towards forming particle–hole pairing or density waves


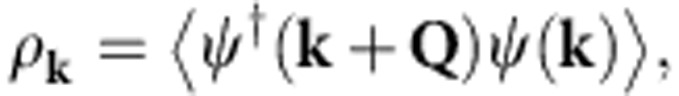


(with 
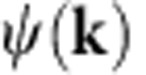
 a Fourier-transformed annihilation operator) is logarithmically divergent due to nesting and the system has generic instabilities with repulsive interactions. With nearest neighbor interactions for spinless fermions, the ground-state density wave order *ρ*_**k**_ is momentum independent, implying a trivial 3D checkerboard pattern in real space. However, for Rydberg-dressed atoms, the interaction range, *r*_c_ can be comparable to several lattice spacings, which then frustrates the checkerboard pattern, giving rise to possibilities of unconventional density waves^26^,^31^.

Within one-loop RG analysis, we find that the strength of density wave instability is determined by the eigenvalue problem of a rescaled interaction matrix *γ* whose symmetry is O_h_ × 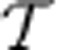 (see Methods and Supplementary Note 1). Different density waves correspond to eigenvectors of *γ*, which are classified according to irreducible representations of the symmetry group^32^,^33^. The symmetry classification of density waves and their representative irreducible basis functions are shown in Table 1. The trivial density wave is 
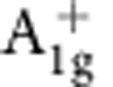
 and all other density waves necessarily have more complicated momentum dependence for symmetry reasons. The latter leads to particle–hole pairing on the bonds in real space^26^.

### Unconventional density wave topology

Before presenting our microscopic results, let us first look at a non-trivial superposed density wave, 
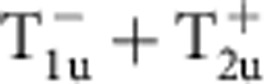
 (see Table 1). We introduce


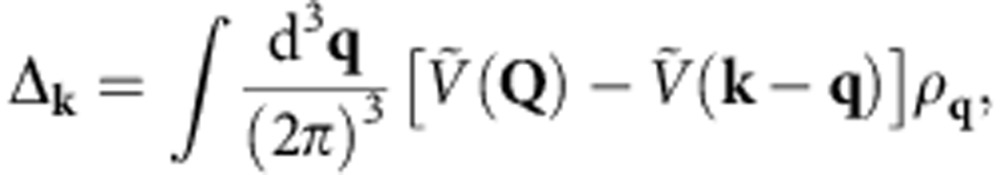


where 
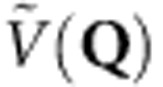
 is the Fourier transform of the interaction *V*(**r**). In the 
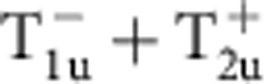
 state, Δ_**k**_ takes a form


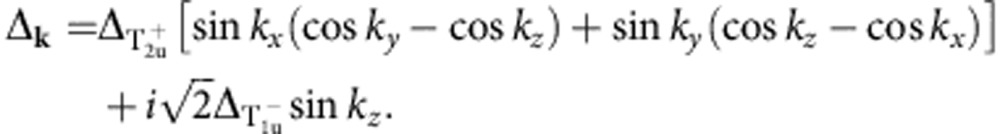


**Figure 1:**
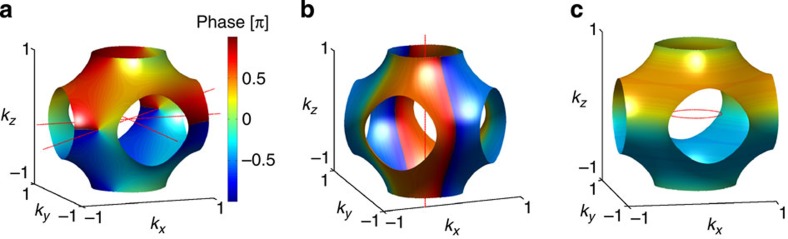
Density wave topology. The colour index shows the phase angle (in units of *π*) of the momentum-dependent density wave order Δ_**k**_ projected on the Fermi surface. (**b**,**c**) use the the same colour scheme as shown in **a**. **a**–**c** correspond to topologically distinct density wave states. The red dotted lines are vortex lines around which the phase of Δ_**k**_ changes by 2*mπ* (*m*≠0). Right on vortex lines, Δ_**k**_ vanishes. In **a**, one vortex line crosses the Fermi surface. In both **a**,**b**, all vortex lines cannot be adiabatically removed without touching the Fermi surface, and thus are topologically robust, whereas in **c**, the vortex line is adiabatically removable and is thus topologically trivial.

Here 
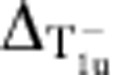
 and 
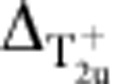
 take real values. Such a density wave order has a rich topological structure in 3D momentum space (Fig. 1a), with three vortex lines located at (0, *l*, 0), (*l*, 0, 0) and (*l*, *l*, 0), with *l*∈[−*π*/*a*, *π*/*a*). These vortex lines are topologically robust in the sense that they cannot be smoothly removed without touching the Fermi surface. The Bogoliubov-de Gennes Hamiltonian to describe quasiparticles in the density wave background reads,


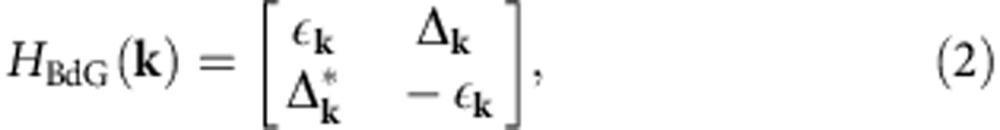


which can be rewritten in terms of Pauli matrices as 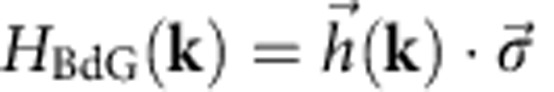. At the points 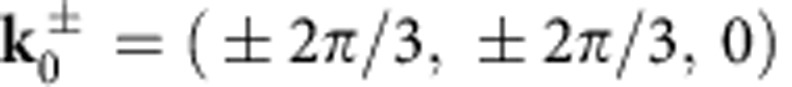 where vortex lines cross the Fermi surface, the magnitude of 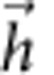 vanishes, leading to zero energy quasiparticles. Around each nodal point, say 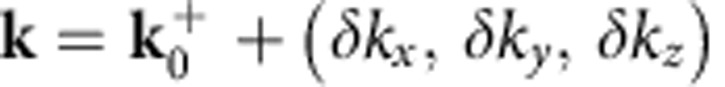, we have 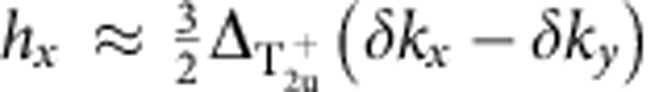 and 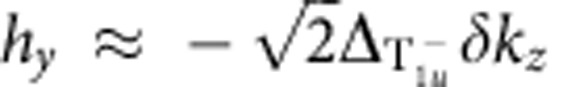. The quasiparticles near the gapless points are thus Weyl fermions^9^,^10^,^11^,^34^,^35^,^36^,^37^ with highly anisotropic velocities. Besides this gapless topological property, it is worth noting that the other two vortex lines that do not cross the Fermi surface are also topologically robust.

In general, we argue that the topology of 3D density waves can be characterized by two integers (*n*_1_,*n*_2_), *n*_1_ being the number of vortex lines that cross the Fermi surface and *n*_2_ the number of other vortex lines that cannot be contracted to one point without touching the Fermi surface. The topological numbers *n*_1_ and *n*_2_ thus encode gapless and gapped topologies, respectively. We emphasize that any two vortex lines connectible by the **Q** vector are equivalent. For gapped density waves, *n*_1_ is necessarily 0, and we have a *Z* classification, whereas for gapless states, both *n*_1_ and *n*_2_ can be finite and the classification is *Z* × *Z*. Using this scheme, the 
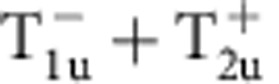
 state has topological numbers (1,2), exhibiting mixed topologies. The density wave topology as constructed is measurable by time-of-flight techniques in atomic experiments (see Methods).

### Phase diagram

**Figure 2:**
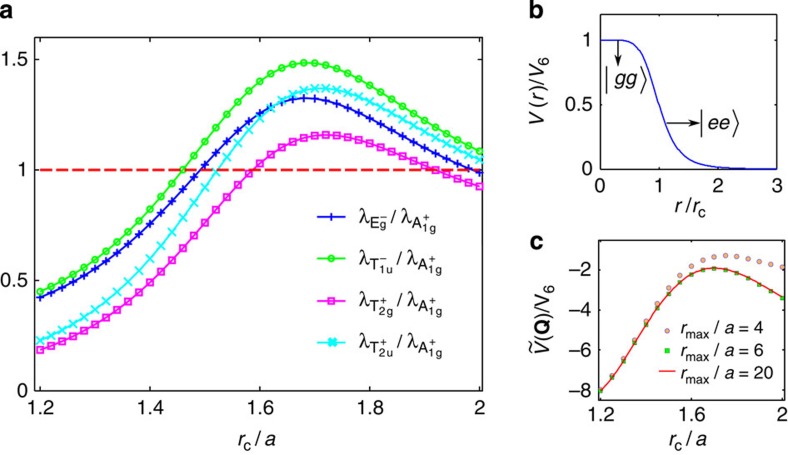
Leading density wave instabilities for Rydberg-dressed atomic Fermi gas. (**a**) Most dominant eigenvalues of the *γ* matrix (see Methods), representing the strengths of particle–hole paring instability of corresponding channels. Increasing *r*_c_, the 
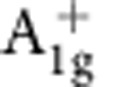
 channel gets suppressed, and other channels, 
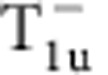
, 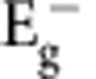, 
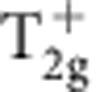
 and 
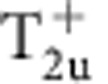
, with non-trivial momentum dependence are enhanced. (**b**) Step-like interaction form for Rydberg-dressed atoms. Two atoms far apart behave as in Rydberg excited states and interact with van der Waals potential, whereas they become more ground state like at short distance. (**c**) The Fourier-transformed interaction 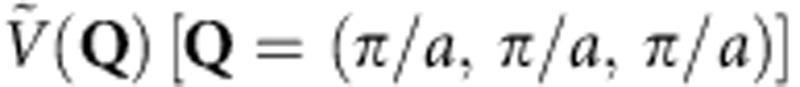. As we increase the cutoff *r*_max_ (see Methods), 
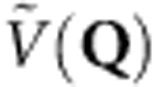
 converges and the truncation error is already negligible when *r*_max_/*r*_c_=3.

We now discuss the actual density waves supported by Rydberg-dressed atoms. With infinitesimal repulsive interaction, the relative strengths of instabilities in different channels are fully determined by the non-locality strength *r*_c_/*a*. The leading instabilities, as quantified by the corresponding eigenvalues of the *γ* matrix, are shown in Fig. 2. When *r*_c_ is small, the interaction is essentially nearest neighbour, for which the *γ* matrix is mainly determined by the momentum independent part of interaction 
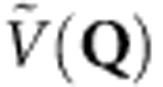
 (the Fourier transform of the interaction at **Q**). This makes the trivial 
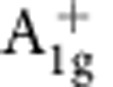
 density wave completely dominant over all other density wave states. Increasing *r*_c_, the longer-ranged part of the interaction becomes more important, which suppresses 
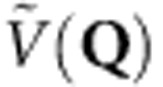
 (see Fig. 2c) and consequently the 
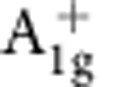
 density wave. Simultaneously, other density wave channels with non-trivial momentum dependence get enhanced. The dominant instabilities are 
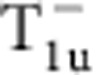
, 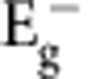, 
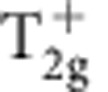
 and 
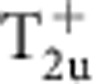
, and we have a transition from 
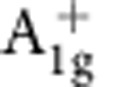
 to 
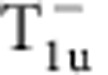
 around *r*_c_/*a*=1.46. The phase diagram based on the instability analysis from RG is suggestive, and is rigorous in leading order. However, considering finite interactions, non-linearity may lead to significant physical effects. In particular, when *r*_c_ reaches the scale of lattice spacing, the instability strengths in different channels are quasi-degenerate, which we attribute to the ‘step-like' feature of the Rydberg-dressed interaction (see Methods). Non-linear effects must then be taken into account to determine the actual phase diagram.

To incorporate non-linearity, we numerically simulate a system of size 64 × 64 × 64 with periodic boundary condition using self-consistent methods (see Methods). The solution for the ground-state density wave order Δ_**k**_ is expanded in terms of basis functions


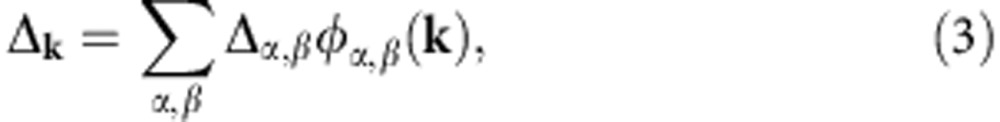


where *φ*_*α*,*β*_(**k**) are the basis functions from symmetry classification (see Table 1), with *α* labelling different classes and *β* different functions within one class. In the self-consistent theory including non-linear effects, spontaneous symmetry breaking could occur and superpositions of density waves from different classes are allowed, subject to the constraint 
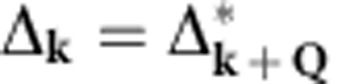
. The coefficients Δ_*α*,*β*_ are required to be real.

**Figure 3:**
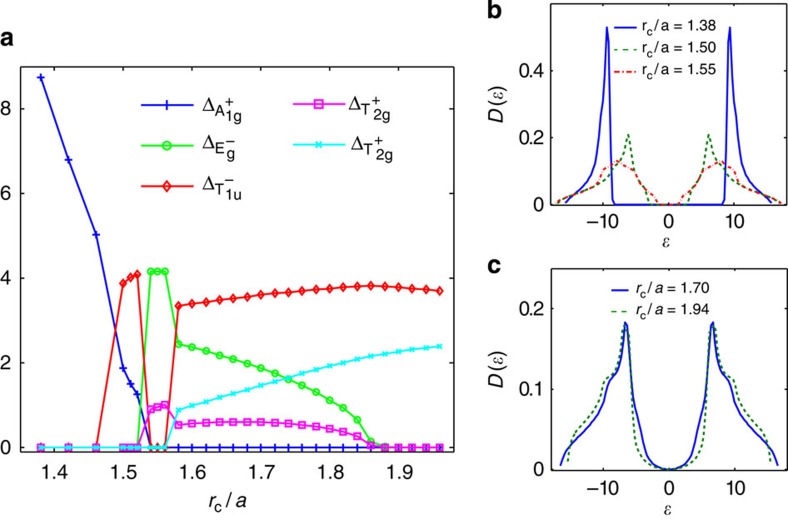
Phase diagram for Rydberg-dressed atomic Fermi gas. In this plot, the interaction strength is fixed to be *V*_6_/*t*=4. To quantify contributions from different classes, we introduce 
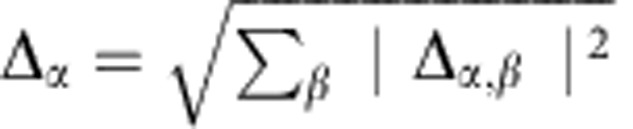
, (see Equation 3). (**a**) The phase diagram with varying Condon radius *r*_c_. As we increase *r*_c_, we get a sequence of density wave phases, 
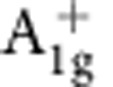
, 
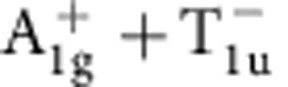
, 
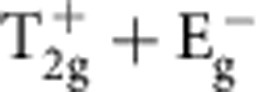
, 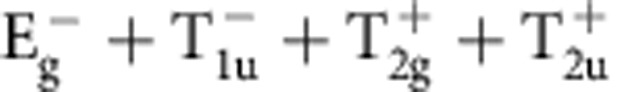 and 
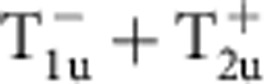
. The first three are fully gapped and the last two are gapless. The phase transition between the two gapless phases is found to be second order. The corresponding broken symmetry is space inversion. The phase transitions among the gapped phases are first order. (**b**,**c**) The density of states for gapped and gapless phases, respectively.

In our self-consistent calculations, spontaneous symmetry breaking of O_h_ × 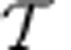 is found for a large range of *r*_c_ and the symmetry-broken ground states support various superposed density waves, yielding a rich phase diagram (Fig. 3). Surprisingly, all the superposed states are found to break 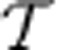 symmetry and consequently the time-reversal symmetry due to the resultant complex structure in Δ_**k**_. The topological gapless density wave 
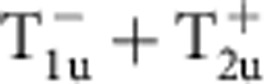
 is indeed an allowed solution as argued earlier. The other gapless phase that occupies a large region in the phase diagram is 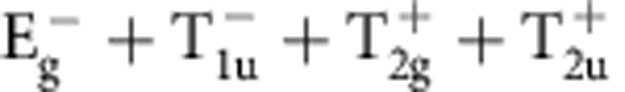. In both gapless phases, the quasiparticle density of states has a ‘soft-gap' feature (Fig. 3c), a signature of the emergent topological Weyl fermions. In addition, we find three gapped phases, 
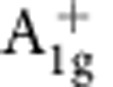
, 
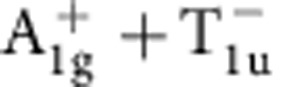
 and 
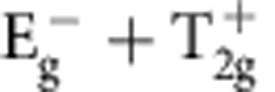
, whose quasiparticle density of states has a ‘hard-gap' (Fig. 3b). In atomic experiments, the quasiparticle spectrum and density of states can be directly measured by spectroscopic techniques^38^,^39^.

We now discuss topological properties of the density waves in the phase diagram. In 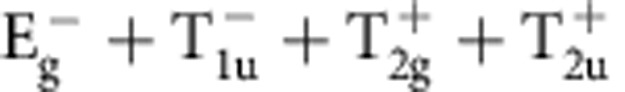 state, as compared with 
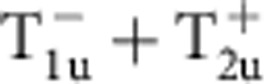
, its complex order Δ_**k**_ gets an additional contribution, which is 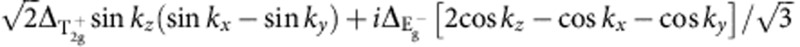. Treating these additional components as perturbations, we get three vortex lines located at 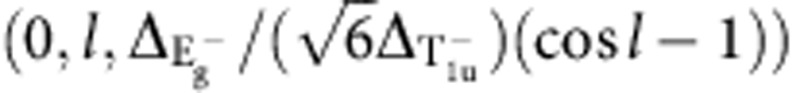, 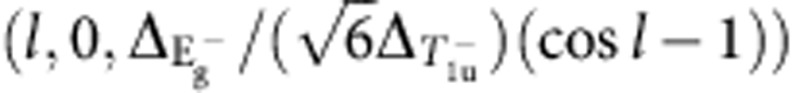 and 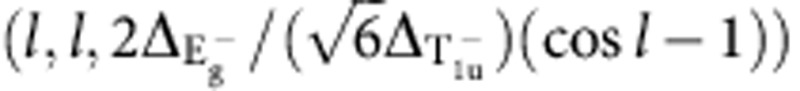, the third of which crosses Fermi surface. These vortex lines are no longer straight, but otherwise have the same topology as shown in Fig. 1a. The 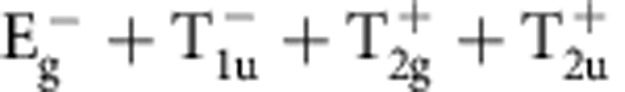 state is topologically equivalent to 
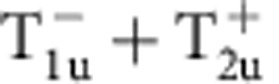
, having the same topological numbers (1,2).

In the 
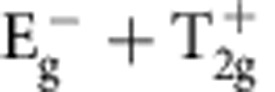
 state which is gapped, Δ_**k**_ takes a form


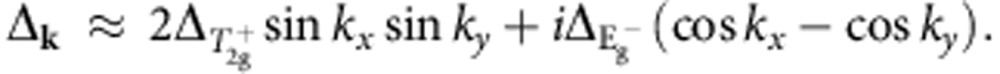


The resulting vortex line (with vorticity 2) is located along the *k*_*z*_ axis (note that the O_h_ symmetry has been spontaneously broken), thus not crossing the Fermi surface (see Fig. 1b). This state has topological numbers (0,2) in our classification scheme. Actually, in this state, the quasiparticle Hamiltonian *H*_BdG_(**k**), with arbitrary fixed *k*_*z*_, has finite Chern number 2. Such a state thus exhibits 3D quantum anomalous Hall effect, featuring chiral surface modes, which mediate highly anisotropic transport properties—dissipationless in *x* or *y* direction but diffusive in the *z* direction^40^,^41^. In the other gapped state, 
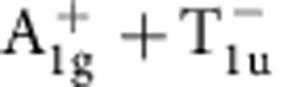
, we have


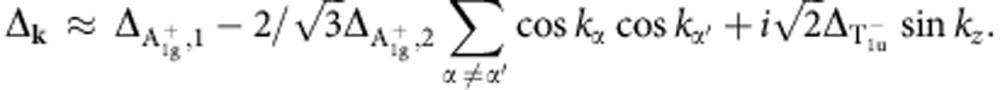


The vortex line of this state is shown in Fig. 1c, but it is topologically unstable. The 
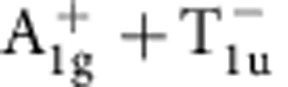
 state has trivial topological numbers (0,0), topologically equivalent to 
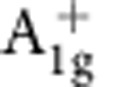
.

## Discussion

We expect our proposed topological density waves to be generic for Rydberg-dressed atomic Fermi gases even if the interaction potential is not precisely of the specific *r*^6^ form. The key qualitative ingredient is the step-like feature in the interaction. To confirm this, we also have carried out calculations for Rydberg *p*-wave-dressed atoms^24^, and similar topological states are indeed found (see Supplementary Fig. 2, Supplementary Table 1 and Supplementary Note 2). We therefore believe that our prediction for novel collective topological density waves should apply generically to dressed Rydberg optical lattices. Besides, by assuming momentum dependence of Fermi velocity is negligible, we even derive an approximate criterion for unconventional density waves to occur in generic long-range interacting systems (see Methods).

Considering the effect of a harmonic potential as present in most atomic experiments, the gapped density wave states are expected to be robust and occupy a finite spatial region as analogous to incompressible Mott shells in trapped lattice Bose gases^42^. For the gapless density waves with emergent Weyl points, we expect to have Weyl fermions of finite density in the trap, where more interesting many-body effects are worth future exploration.

## Methods

### Density wave instability under infinitesimal repulsion

The single-particle energy dispersion for the 3D cubic lattice with O_h_ symmetry is 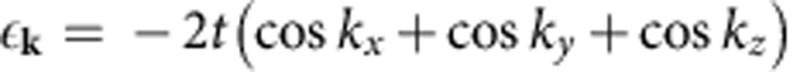. Having a non-local density–density interaction, the scattering vertex among single-particle modes **k**_1_, **k**_2_, **k**_3_ and **k**_4_ is


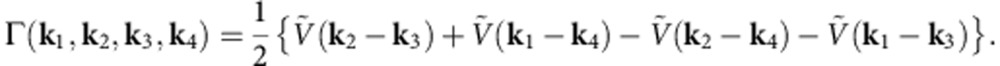


At half filling, from the Fermi surface nesting, repulsive interactions cause instabilities in density wave channels. Such instabilities can be tracked by RG flow of the effective couplings near the Fermi surface,


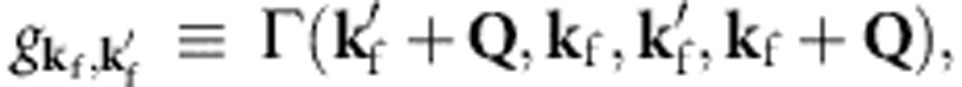


The RG equation at one-loop level^43^,^44^ reads


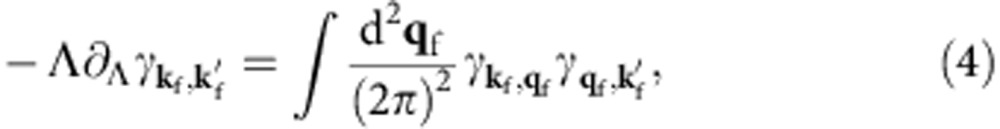


with Λ the momentum cutoff of the theory and *γ* a rescaled interaction matrix,


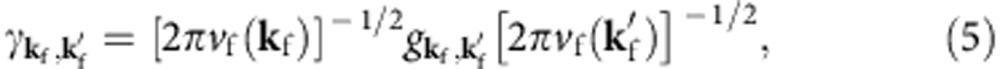


where *v*_f_(**k**_f_) is the Fermi velocity. In the RG flow, the largest eigenvalue of the *γ* matrix diverges most quickly as approaching the low energy limit. The magnitudes of eigenvalues quantify the relative strength of instabilities in different channels. The symmetry group of *γ* is O_h_ × 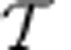, with *T* a two-element group involving the transformation *T*(**k**)=**k**+**Q**.

### Non-linear effects and self-consistent theory

The variational state we choose to describe density wave order^26^ is


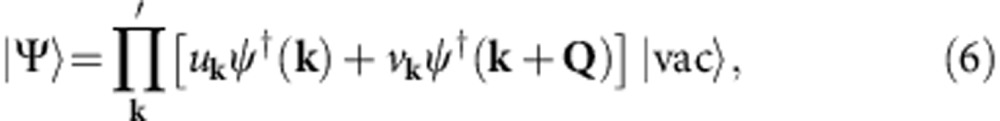


with |vac〉 the vacuum and **k** running over one half of the Brillouin zone, for example, *k*_*z*_⩾0. The self-energy and density wave order are given by (see Supplementary Note 1)


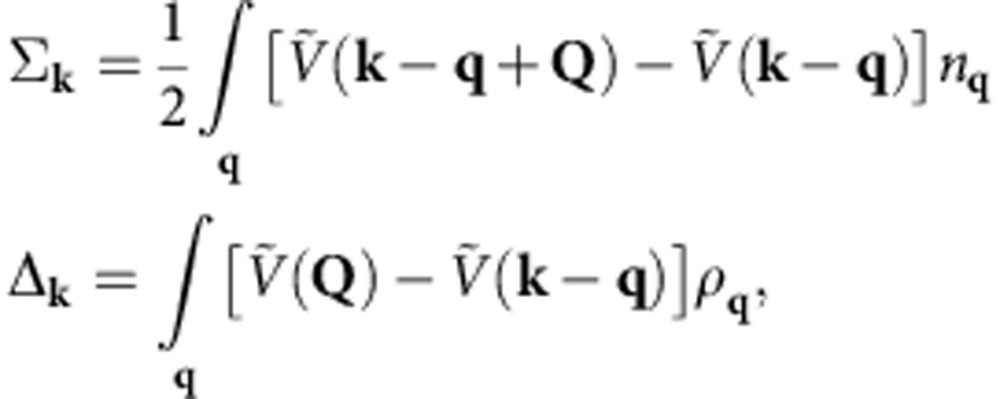


with 
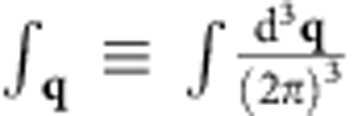
, 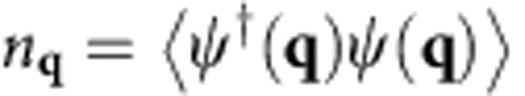 and 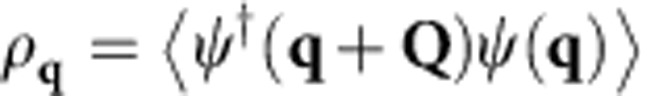. The self-consistent equations are obtained to be


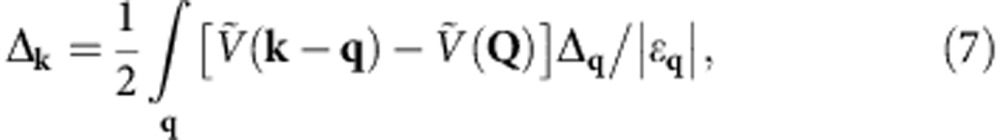



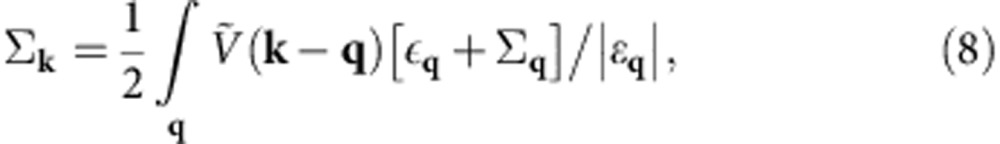


with quasiparticle spectra 
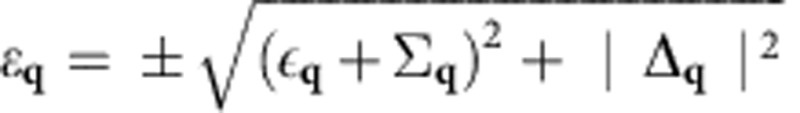
. With moderate interaction strength, self-energy corrections could be significant, but the modification to the Fermi surface is reasonably weak (see Supplementary Fig. 1).

In numerics, a brute force treatment of self-consistent iteration would lead to numerical cost of 
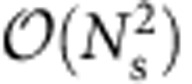
, with *N*_s_ the number of lattice sites, which makes the numerical calculations too heavy for large systems. We simplify the problem by rewriting


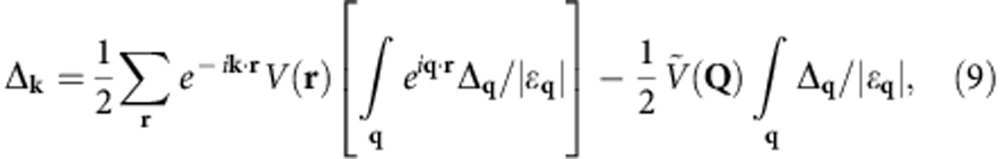



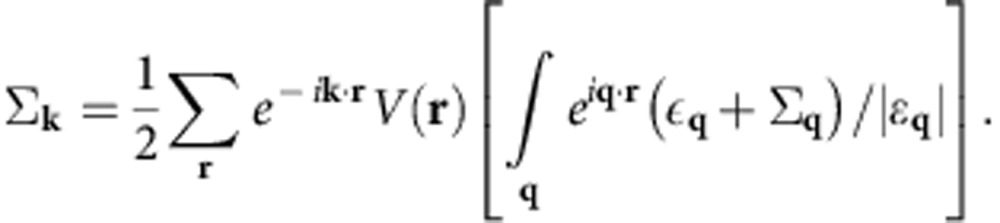


Following the bracketing order above in numerical iteration, the cost is greatly reduced. In our calculations, we choose a long-range cutoff *r*_max_, so that *V*(|**r**|>*r*_max_) is set to be 0. The numerical cost for each iteration is then 
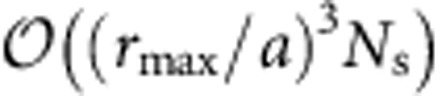
, which makes it feasible to simulate large systems. For all numerical results presented in the main text, *r*_max_ is set to be 6 × *a* and varied to ensure that results do not change qualitatively.

### An approximate criterion for unconventional density waves

Since solving the eigenvalue problem of the *γ* matrix requires numerics in general, we would like to give an approximate criterion for unconventional density waves to occur in a generic system, and give some intuition why such orders are supported by Rydberg-dressed interactions. The assumption we take here is that the momentum dependence of Fermi velocity is negligible. Under this assumption, the eigenvalue problem of *γ* becomes equivalent to that of


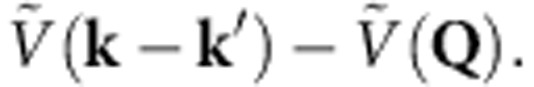


From Fourier transformation, we know


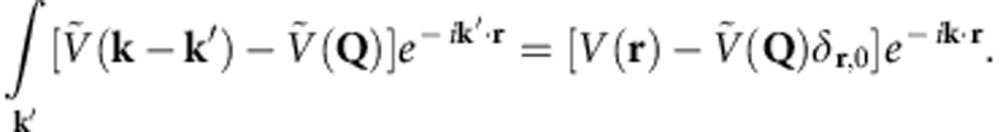


The density wave channels (Δ_**k**_) are then given by the plane waves *e*^−**i***k*·**r**^, **r** labelling different solutions. The eigenvalue associated with the trivial density wave with **r**=0 is then 
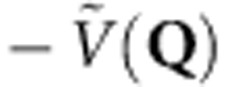
. For other density waves having momentum dependence, with **r**≠0, the eigenvalues are *V*(**r**). With isotropic interactions, the density wave solutions with the same |**r**| are degenerate. In particular with step-like interactions as for Rydberg-dressed atoms, the solutions with |**r**|<*r*_c_ are actually all quasi-degenerate. This would strongly amplify non-linear effects, giving rise to possibilities for superposed density waves.

The criterion for unconventional density waves to dominate over the trivial one is,


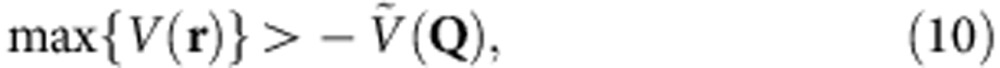


which implies for the Rydberg interaction that


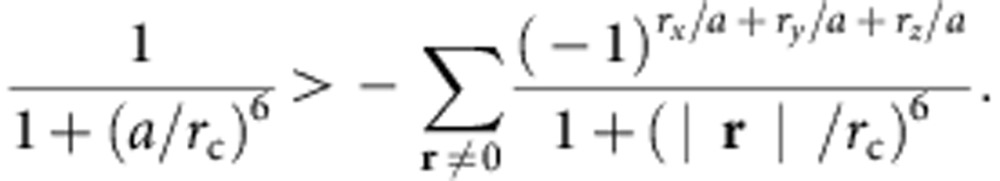


The parameter region where we find interesting phases in the full RG analysis and self-consistent calculations, indeed satisfies the above condition. For general interactions, the most likely region to look for unconventional density waves can be easily identified by the derived criterion in equation (10).

### Rydberg dressing scheme and experimental accessibility

Coherently coupling ground-state (|*g*〉) atoms to Rydberg excitations (|*e*〉), the internal dynamics of one single atom is described by a Hamiltonian


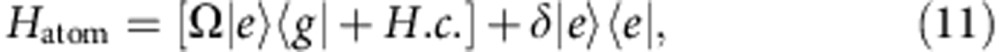


with Rabi frequency Ω and detuning *δ*^19^,^21^,^22^,^24^,^25^. Atoms in Rydberg states interact through a strong van der Waals interaction, 
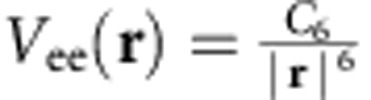
. With far off-resonant atom-photon coupling, Ω/*δ*≪1, ground-state atoms get weakly admixed with Rydberg states, which yields an effective interaction as shown in Fig. 2b. This scheme, unlike resonantly populating Rydberg states, has been theoretically shown to provide long life-time (from a few to hundreds ms) atomic samples^21^,^22^. We take a lattice spacing *a* of 500 nm. For ^40^K atoms in Rydberg *s*-states with a principal quantum number *n*=25, the bare van der Waals interaction strength is *C*_6_/*a*^6^≈2*π* × 100 MHz (ref. 45). With a detuning *δ*=2*π* × 50 MHz, and Rabi frequency Ω=2*π* × 5 MHz, we have *r*_c_≈*a* and *V*_6_≈2*π* × 10 kHz; and with *δ*=2*π* × 2 MHz and Ω=2*π* × 0.4 MHz, we have *r*_c_≈1.7*a* and *V*_6_≈2*π* × 6 kHz. The resultant interaction strengths are comparable to typical nearest neighbor tunnelling in optical lattices (on the order of a few kHz). We expect the equilibration time to be set by the collision time (around a few tenth ms) from previous study on non-equilibrium Fermi–Hubbard models^46^. Our predicted mixed topological density waves are in principle accessible with the off-resonant dressing scheme, although the required laser power has not yet been achieved in the present experiment^19^. The transition temperatures can be estimated from the energy gap (or the ‘soft-gap' for gapless states) which is around 100 nK, and rigorous calculations are left for future works. We want to mention here that the parameter region *r*_c_≪*a*, which could be more experimentally feasible, is expected to also support superposed density waves (equation 10), although self-consistent calculations there are more challenging.

### Experimental signature of density wave topology

In experiments, the momentum dependence of the density wave order can be extracted from time-of-flight measurements. Using mean field theory, it is straightforward to show that


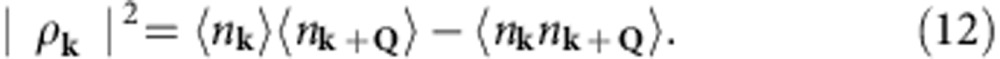


At finite temperature, to extract |*ρ*_**k**_|^2^, it is necessary to measure both the product 〈*n*_**k**_〉〈*n*_**k**+**Q**_〉 and the correlation 〈*n*_**k**_ *n*_**k**+**Q**_〉. At half filling, the latter originates purely from thermal fluctuations. The former is dominant when the temperature is not too high as compared with the quasiparticle energy gap (or the ‘soft-gap' for gapless states). This implies that it should be experimentally straightforward to get a precise measurement of |*ρ*_**k**_|^2^. In the zero temperature limit, the density wave order is completely determined by the product |*ρ*_**k**_|^2^=〈*n*_**k**_〉〈*n*_**k**+**Q**_〉. Moreover from the relation,


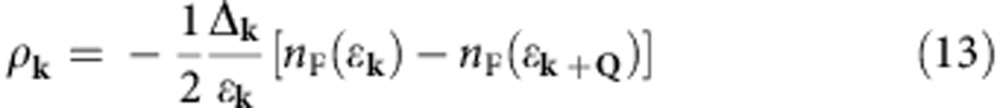


the vortex lines of Δ_**k**_ immediately follow by tracking the vanishing points of |*ρ*_**k**_|. With vortex lines located in the 3D momentum space, the topological numbers of density waves can be easily extracted.

## Additional information

**How to cite this article**: Li, X. & Das Sarma, S. Exotic topological density waves in cold atomic Rydberg-dressed fermions. *Nat. Commun.* 6:7137 doi: 10.1038/ncomms8137 (2015).

## Supplementary Material

Supplementary Information Supplementary Figures 1-2, Supplementary Tables 1, Supplementary Notes 1-2, Supplementary References

## Figures and Tables

**Table 1:** Symmetry classification of three-dimensional density wave orders. The classification is according to irreducible representation of the symmetry group O_h_ × 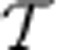 .

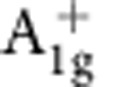	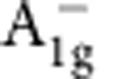	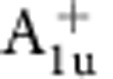	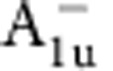
1,*cos k*_*x*_cos *k*_*y*_+cos *k*_*y*_cos *k*_*z*_+cos *k*_*z*_cos *k*_*x*_,	*i*(cos *k*_*x*_+cos *k*_*y*_+cos *k*_*z*_)	—	—
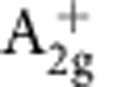	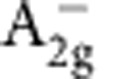	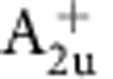	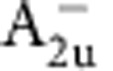
—	—	—	—
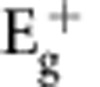	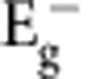	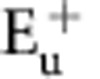	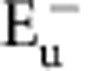
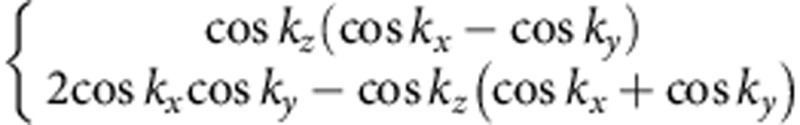	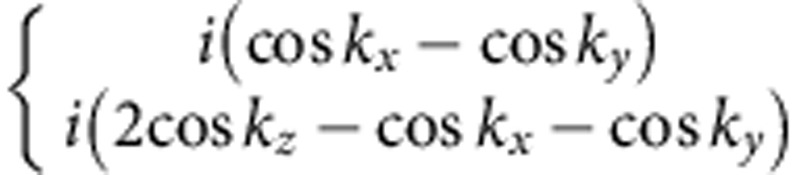	—	—
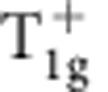	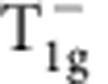	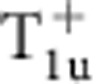	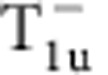
—	—	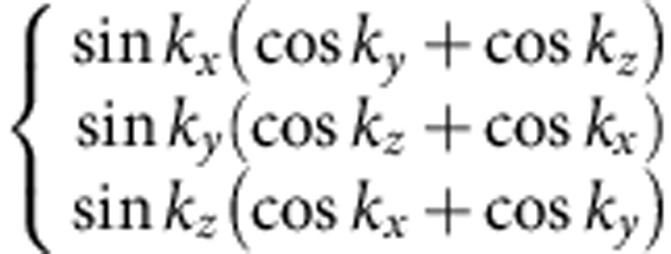	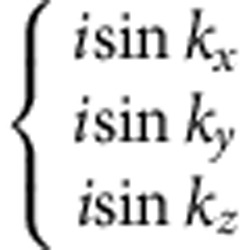
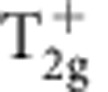	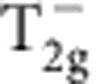	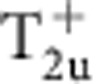	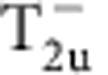
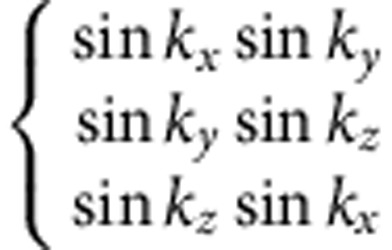	—	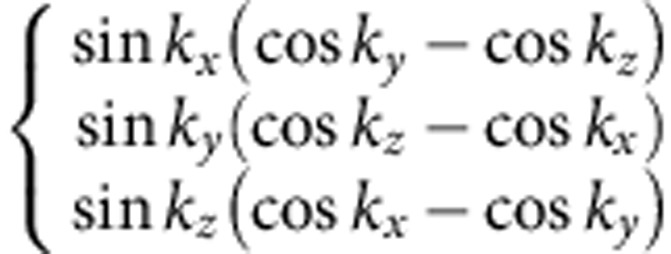	—

In the labelling of different classes, the superscript ± tells how the density wave transforms under 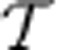 symmetry. The basis functions representing particle–hole paring of upto next nearest neighbouring sites are given. Owing to the constraint 
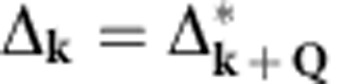
, the basis functions in 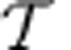 even (odd) classes are real (purely imaginary). The normalization constants for the basis functions are neglected in this table.
