# Supplementary material for: Exotic topological density waves in cold atomic Rydberg-dressed fermions
Source: Nat Commun. 2015 May 14;6:7137. doi: 10.1038/ncomms8137 (PMC4478999; doi:10.1038/ncomms8137)
Supplement: Supplementary Information — Supplementary Figures 1-2, Supplementary Tables 1, Supplementary Notes 1-2, Supplementary References [file ncomms8137-s1.pdf]

# Supplementary Figures

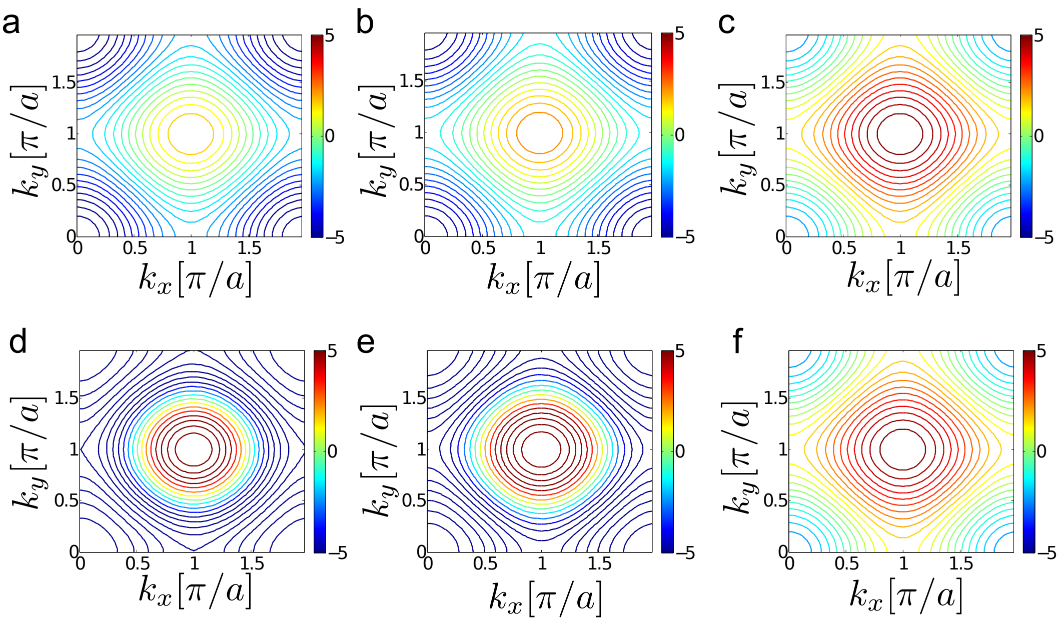


**Supplementary Figure 1**: Illustration of self-energy corrections. In this plot we choose $r_c/a = 1.86$ and $V_6/t = 4$. **a**, **b** and **c** show contour plots of bare dispersion $\epsilon_{\mathbf{k}}$ with $k_z a = 0$, $\frac{\pi}{4}$ and $\frac{3\pi}{4}$, respectively. **d**, **e** and **f** show $\epsilon_{\mathbf{k}} + \Sigma_{\mathbf{k}}$ for the same set of $k_z$. Although self-energy corrections are significant, its modification to the Fermi surface (defined by $\epsilon_{\mathbf{k}} + \Sigma_{\mathbf{k}} = 0$) is weak. The property holds for other choice of parameters as well.

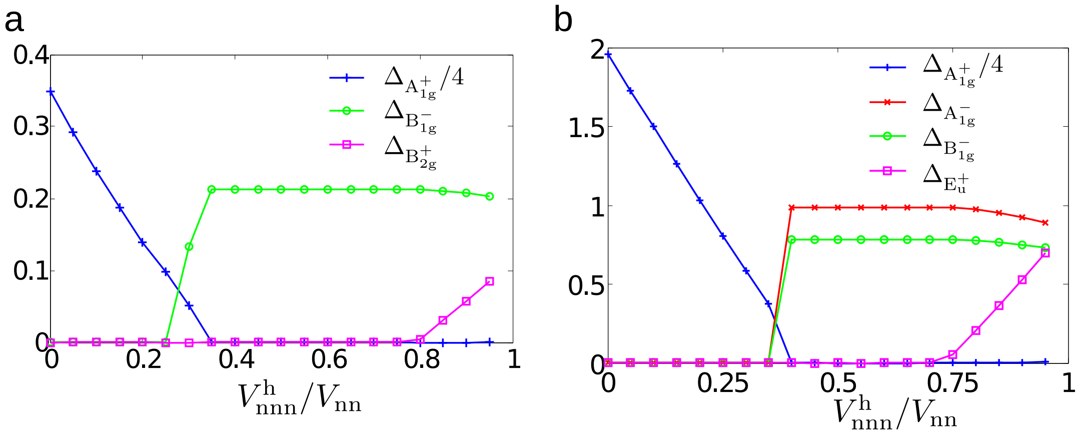


**Supplementary Figure 2**: Phase diagram from self-consistent calculations with for $p$-wave Rydberg dressed atoms. In this plot we choose $V_{\mathrm{nn}}/t_z = 2$, $V_{\mathrm{nnn}}^z/V_{\mathrm{nn}} = 0.2$. **a** and **b** show the symmetric ($t_z = t_x = t_y$) and asymmetric ($t_z = 4t_x = 4t_z$) tunneling cases, respectively. The $\mathrm{A}_{1g}^+$ component $\Delta_{\mathrm{A}_{1g}^+}$ is rescaled by a factor 4 for illustration purposes.

# Supplementary Tables

| | | | |
|---|---|---|---|
| $\mathrm{A}_{1g}^{+}$ | $\mathrm{A}_{1g}^{-}$ | $\mathrm{A}_{1u}^{+}$ | $\mathrm{A}_{1u}^{-}$ |
| $1,\ \cos k_x \cos k_y,\ (\cos k_x + \cos k_y)\cos k_z$ | $i(\cos k_x + \cos k_y),\ i\cos k_z$ | $(\cos k_x + \cos k_y)\sin k_z$ | $i \sin k_z$ |
| $\mathrm{E}_{g}^{+}$ | $\mathrm{E}_{g}^{-}$ | $\mathrm{E}_{u}^{+}$ | $\mathrm{E}_{u}^{-}$ |
| $\begin{cases} \sin k_x \sin k_z \\ \sin k_y \sin k_z \end{cases}$ | | $\begin{cases} \sin k_x \cos k_y,\ \sin k_x \cos k_z \\ \cos k_x \sin k_y,\ \sin k_y \cos k_z \end{cases}$ | $\begin{cases} i\sin k_x \\ i \sin k_y \end{cases}$ |
| $\mathrm{B}_{1g}^{+}$ | $\mathrm{B}_{1g}^{-}$ | $\mathrm{B}_{1u}^{+}$ | $\mathrm{B}_{1u}^{-}$ |
| $(\cos k_x - \cos k_y)\cos k_z$ | $i(\cos k_x - \cos k_y)$ | $(\cos k_x - \cos k_y)\sin k_z$ | |
| $\mathrm{B}_{2g}^{+}$ | $\mathrm{B}_{2g}^{-}$ | $\mathrm{B}_{2u}^{+}$ | $\mathrm{B}_{2u}^{-}$ |
| $\sin k_x \sin k_y$ | | | |

**Supplementary Table 1** : Classification of density wave orders for non-local interactions up to next nearest neighbor according to irreducible representation of the symmetry group $\mathrm{D}_{4h} \times \mathcal{T}$.

# Supplementary Notes

### Supplementary Note 1. A CONSISTENT THEORY FOR DENSITY WAVE ORDER

The model Hamiltonian to describe a Rydberg dressed atomic Fermi gas in a three dimensional (3d) cubic optical lattice is $H = H_0 + H_{\rm int}$ with

$$H_0 = \sum_{\mathbf{r},\alpha} \left[ -t_\alpha c^\dagger_{\mathbf{r}} c_{\mathbf{r}+a\hat{e}_\alpha} + h.c. \right],$$
$$H_{\rm int} = \frac{1}{2} \sum_{\mathbf{r},\mathbf{r}'} V(\mathbf{r}-\mathbf{r}') c^\dagger_{\mathbf{r}} c^\dagger_{\mathbf{r}'} c_{\mathbf{r}'} c_{\mathbf{r}}.$$

Here $a$ is the lattice constant, $\alpha$ index the three directions $x$, $y$ and $z$, and $c_{\mathbf{r}}$ the fermionic annihilation operator positioned at $\mathbf{r}$. The atomic interaction potential, assuming the Rydberg state is $s$-wave, takes an isotropic form, $V(\mathbf{r}) = V_6/[1+(|\mathbf{r}|/r_c)^6]$, for $r \neq 0$. For fermionic atoms considered here, $V(0)$ is set to be zero.

To describe a density wave ground state, we take a variational state

$$|\Psi\rangle = \prod\nolimits'_{\mathbf{k}} \left[ u_{\mathbf{k}} c^\dagger_{\mathbf{k}} + v_{\mathbf{k}} c^\dagger_{\mathbf{k}+\mathbf{Q}} \right] |\text{vac}\rangle.$$

Here $|\text{vac}\rangle$ is the vacuum, and we have a normalization condition $|u_{\mathbf{k}}|^2 + |v_{\mathbf{k}}|^2 = 1$. In $\prod'_{\mathbf{k}}$, $\mathbf{k}$ runs over one half of the Brillouin zone, say $k_z \geq 0$. We can define $u_{\mathbf{k}}$ and $v_{\mathbf{k}}$ in the whole Brillouin zone by the relation $u_{\mathbf{k}+\mathbf{Q}} = v_{\mathbf{k}}$, $v_{\mathbf{k}+\mathbf{Q}} = u_{\mathbf{k}}$. Half filling is already enforced within this variational ansatz. The variational energy cost $E = \langle \Psi | H_0 + H_{\rm int} | \Psi \rangle$ is obtained to be

$$E = -\frac{1}{2} \int \frac{d^3\mathbf{k}}{(2\pi)^3} \sqrt{(\epsilon_{\mathbf{k}} + \Sigma_{\mathbf{k}})^2 + |\Delta_{\mathbf{k}}|^2}$$
$$+\frac{1}{2} \int \frac{d^3\mathbf{k}}{(2\pi)^3} \frac{d^3\mathbf{k}'}{(2\pi)^3} \Delta^*_{\mathbf{k}} \tilde{V}^{-1}_{\mathbf{Q}}(\mathbf{k}-\mathbf{k}') \Delta_{\mathbf{k}'},$$

with $\tilde{V}_{\mathbf{Q}}(\mathbf{k}-\mathbf{k}') = \tilde{V}(\mathbf{k}-\mathbf{k}') - \tilde{V}(\mathbf{Q})$. Minimizing the energy, we get the Bogoliubov-de Gennes (BdG) equation

$$H_{\rm BdG}(\mathbf{k}) \begin{bmatrix} u_{\mathbf{k}} \\ v_{\mathbf{k}} \end{bmatrix} = \varepsilon_{\mathbf{k}} \begin{bmatrix} u_{\mathbf{k}} \\ v_{\mathbf{k}} \end{bmatrix},$$

with

$$H_{\rm BdG}(\mathbf{k}) = \begin{bmatrix} \epsilon_{\mathbf{k}} + \Sigma_{\mathbf{k}} & \Delta_{\mathbf{k}} \\ \Delta^*_{\mathbf{k}} & -\epsilon_{\mathbf{k}} - \Sigma_{\mathbf{k}} \end{bmatrix},$$

where the self-energy is

$$\Sigma_{\mathbf{k}} = \frac{1}{2} \int \frac{d^3\mathbf{q}}{(2\pi)^3} \left[ \tilde{V}(\mathbf{k}-\mathbf{q}+\mathbf{Q}) - \tilde{V}(\mathbf{k}-\mathbf{q}) \right] |u_{\mathbf{q}}|^2$$

and the off-diagonal term is the density wave order,

$$\Delta_{\mathbf{k}} = \int \frac{d^3\mathbf{q}}{(2\pi)^3} \left[\tilde{V}(\mathbf{Q}) - \tilde{V}(\mathbf{k}-\mathbf{q})\right] u_{\mathbf{q}} v^*_{\mathbf{q}}.$$

The chemical potential has been shifted by $\frac{1}{2}\tilde{V}(0)$ to compensate Hartree corrections to the self-energy. By definition, the density wave order satisfies $\Delta_{\mathbf{k}} = \Delta^*_{\mathbf{k}+\mathbf{Q}}$, which is an important difference of particle-hole pairing from particle-particle paring in superconducting states. Diagonalizing the BdG Hamiltonian, quasiparticle energies are given as $\varepsilon_{\mathbf{k}} = \pm\sqrt{(\epsilon_{\mathbf{k}} + \Sigma_{\mathbf{k}})^2 + |\Delta_{\mathbf{k}}|^2}$. In variational calculations, we choose the negative branch, and the self-consistent equations are then obtained to be

$$\Delta_{\mathbf{k}} = \frac{1}{2}\int \frac{d^3\mathbf{q}}{(2\pi)^3} \left[\tilde{V}(\mathbf{k}-\mathbf{q}) - \tilde{V}(\mathbf{Q})\right] \Delta_{\mathbf{q}}/|\varepsilon_{\mathbf{q}}|,$$

$$\Sigma_{\mathbf{k}} = \frac{1}{2}\int \frac{d^3\mathbf{q}}{(2\pi)^3} \tilde{V}(\mathbf{k}-\mathbf{q}) \left[\epsilon_{\mathbf{q}} + \Sigma_{\mathbf{q}}\right]/|\varepsilon_{\mathbf{q}}|. \qquad \text{(Supplementary Equation 1)}$$

The effective potential for $\Delta_{\mathbf{k}}$, defined by $U[\Delta_{\mathbf{k}}] = \left(E[\Delta_{\mathbf{k}}] - E[0]\right)/N_s$, reads

$$U[\Delta_{\mathbf{k}}] = -\frac{1}{2}\int \frac{d^3\mathbf{k}}{(2\pi)^3} \left\{\sqrt{(\epsilon_{\mathbf{k}} + \Sigma_{\mathbf{k}})^2 + |\Delta_{\mathbf{k}}|^2} - |\epsilon_{\mathbf{k}} + \Sigma_{\mathbf{k}}|\right\} + \frac{1}{2}\int \frac{d^3\mathbf{k}}{(2\pi)^3}\frac{d^3\mathbf{k}'}{(2\pi)^3} \Delta^*_{\mathbf{k}} \tilde{V}^{-1}_{\mathbf{Q}}(\mathbf{k}-\mathbf{k}')\Delta_{\mathbf{k}'}.$$

With weak interaction, the density wave order $\Delta_{\mathbf{k}}$ is restricted to the region near the Fermi surface. We thus rewrite the momentum in terms of Fermi momentum and the distance from the Fermi surface as

$$\mathbf{k} = \mathbf{k}_{\mathrm{f}} + l e_{\perp},$$

where $e_{\perp}$ is a unit vector perpendicular to the Fermi surface. The effective potential now reads

$$U[\Delta_{\mathbf{k}}] = -\frac{1}{2}\int \frac{d^2\mathbf{k}_{\mathrm{f}}}{(2\pi)^2}\int_0^{\Lambda} \frac{dl}{2\pi}\left[\sqrt{v_{\mathrm{f}}^2(\mathbf{k}_{\mathrm{f}})l^2 + |\Delta_{\mathbf{k}_{\mathrm{f}}}|^2} - v_{\mathrm{f}}(\mathbf{k}_{\mathrm{f}})l\right] + \frac{1}{2}\int \frac{d^2\mathbf{k}_{\mathrm{f}}}{(2\pi)^2}\frac{d^2\mathbf{k}'_{\mathrm{f}}}{(2\pi)^2}\Delta^*_{\mathbf{k}_{\mathrm{f}}} g^{-1}_{\mathbf{k}_{\mathrm{f}}\mathbf{k}'_{\mathrm{f}}}\Delta_{\mathbf{k}'_{\mathrm{f}}},$$

where $\Lambda$ is a high-momentum cutoff, and the coupling matrix $[g]$ is the matrix $[\tilde{V}_{\mathbf{Q}}]$ projected onto the Fermi surface. To see the momentum dependence of the $\Delta_{\mathbf{k}}$, we expand $U[\Delta_{\mathbf{k}}]$ to the second order as,

$$U[\Delta] \approx -\frac{1}{4\pi}\ln\left(\frac{\Lambda}{\tau}\right)\int \frac{d^2\mathbf{k}_{\mathrm{f}}}{(2\pi)^2}\frac{|\Delta_{\mathbf{k}_{\mathrm{f}}}|^2}{v_{\mathrm{f}}(\mathbf{k}_{\mathrm{f}})} + \frac{1}{2}\int \frac{d^2\mathbf{k}_{\mathrm{f}}}{(2\pi)^2}\frac{d^2\mathbf{k}'_{\mathrm{f}}}{(2\pi)^2}\left[\frac{\Delta^*_{\mathbf{k}_{\mathrm{f}}}}{\sqrt{v_{\mathrm{f}}(\mathbf{k}_{\mathrm{f}})}}\right]\sqrt{v_{\mathrm{f}}(\mathbf{k}_{\mathrm{f}})} g^{-1}_{\mathbf{k}_{\mathrm{f}}\mathbf{k}'_{\mathrm{f}}}\sqrt{v_{\mathrm{f}}(\mathbf{k}'_{\mathrm{f}})}\left[\frac{\Delta_{\mathbf{k}'_{\mathrm{f}}}}{\sqrt{v_{\mathrm{f}}(\mathbf{k}'_{\mathrm{f}})}}\right],$$

where an infrared cutoff $\tau$ is introduced to regularize the perturbative expansion. This scale $\tau$ can be physically identified to be temperature. Minimizing the effective potential, $\frac{\Delta_{\mathbf{k}_{\mathrm{f}}}}{\sqrt{v_{\mathrm{f}}(\mathbf{k}_{\mathrm{f}})}}$ is an eigenvector of the matrix

$$\sqrt{v_{\mathrm{f}}(\mathbf{k}_{\mathrm{f}})} g^{-1}_{\mathbf{k}_{\mathrm{f}},\mathbf{k}'_{\mathrm{f}}}\sqrt{v_{\mathrm{f}}(\mathbf{k}'_{\mathrm{f}})} \equiv \gamma^{-1}_{\mathbf{k}_{\mathrm{f}}\mathbf{k}'_{\mathrm{f}}} \qquad \text{(Supplementary Equation 2)}$$

with the minimal eigenvalue, or equivalently the eigenvector of $\gamma_{\mathbf{k}_{\mathrm{f}}\mathbf{k}_{\mathrm{f}}'}$ with the maximal eigenvalue $\lambda_{\mathrm{max}}$. The corresponding transition temperature is estimated to be

$$\tau_{\mathrm{c}} = \Lambda \exp(-2\pi\lambda^{-1}_{\mathrm{max}}),$$

which is valid for infinitesimal interactions.

It is worth noting that the couplings $g_{\mathbf{k}_{\mathrm{f}}\mathbf{k}'_{\mathrm{f}}}$ actually depend on the cutoff $\Lambda$. The effective potential $U[\Delta]$ is, on the other hand, independent of $\Lambda$ [1], i.e.,

$$\Lambda\partial_{\Lambda} U[\Delta_{\mathbf{k}}] = 0,$$

from which the renormalization group $\beta$-function follows

$$-\Lambda\partial_{\Lambda} g_{\mathbf{k}_{\mathrm{f}}\mathbf{k}'_{\mathrm{f}}} = \int \frac{d^2\mathbf{q}_{\mathrm{f}}}{(2\pi)^2} g_{\mathbf{k}_{\mathrm{f}}\mathbf{q}_{\mathrm{f}}}[2\pi v_{\mathrm{f}}(\mathbf{q}_{\mathrm{f}})]^{-1} g_{\mathbf{q}_{\mathrm{f}}\mathbf{k}'_{\mathrm{f}}}. \qquad \text{(Supplementary Equation 3)}$$

This renormalization equation can also be derived by momentum shell scheme [2]. Analyzing the renormalization group flow, the momentum dependence of the density wave order can be determined from leading divergent channels in the renormalization group flow, analogous to the procedure of extracting dominant channels in unconventional superconductivity [3, 4]. This approach is equivalent to minimizing the effective potential at quadratic order.

## Supplementary Note 2. DENSITY WAVES WITH RYDBERG $p$-WAVE DRESSED ATOMS

In this section we give density wave phases supported by $p$-wave dressed Rydberg atoms [5], whose interaction takes an anisotropic form

$$V(\mathbf{r} \neq 0) = \frac{V_6}{1 + (|\mathbf{r}|/r_c)^6} \sin^4(\theta),$$

with $\theta$ the polar angle. The symmetry group to classify density waves in this system is then $D_{4h} \times \mathcal{T}$. The symmetry classification is shown in Supplementary Table **1** .

For simplicity, we truncate the long-range part of the interaction by taking $r_{\max} = \sqrt{2} \times a$. The interaction is then modeled to be

$$\begin{aligned} V(\pm\hat{x}) &= V(\pm\hat{y}) = V_{\rm nn} \\ V(\pm\hat{x} \pm \hat{y}) &= V_{\rm nnn}^{\rm h} \\ V(\pm\hat{x} \pm \hat{z}) &= V(\pm\hat{y} \pm \hat{z}) = V_{\rm nnn}^{z}. \end{aligned}$$

All other terms are taken to vanish. With tunability of the lattice geometries, the ratio $V_{\rm nnn}^z/V_{\rm nn}$ and $V_{\rm nnn}^{\rm h}/V_{\rm nn}$ is largely tunable between 0 and 1. In this model, the approximate criterion (equation 10 in the main text) for unconventional density waves implies

$$3V_{\rm nn} < 4V_{\rm nnn}^{\rm h} + 8V_{\rm nnn}^{z}. \qquad \text{(Supplementary Equation 4)}$$

We then numerically solve self-consistent equations for $p$-wave Rydberg dressed atoms. Both symmetric ( $t_z = t_x = t_y$) and asymmetric ($t_z = 4t_x = 4t_y$) tunneling cases are studied.

For the symmetric case (see Supplementary Figure **2**a), we find $A_{1g}^+$, $A_{1g}^+ + B_{1g}^-$, $B_{1g}^-$ and $B_{1g}^- + B_{2g}^+$ density waves. The Fermi surface is fully gapped out for both $A_{1g}^+$ and $A_{1g}^+ + B_{1g}^-$ states. In the $B_{1g}^-$ state the Fermi surface is only partially gapped out, and we have gapless lines (protected by $\mathcal{T}$ symmetry), from which the density of states at low energy has linear behavior, i.e., $D(\varepsilon) \propto \varepsilon$. In the $B_{1g}^- + B_{2g}^+$ state, the Fermi surface for this state is fully gapped out, and the complex order $\Delta_{\mathbf{k}}$ has a vortex line (with vorticity 2) elongated along the $k_z$ axis. This $B_{1g}^- + B_{2g}^+$ state is topologically equivalent to the discussed $E_g^- + T_{2g}^+$ state in the main text.

For the asymmetric case (see Supplementary Figure **2**b), we find $A_{1g}^+$, $A_{1g}^- + B_{1g}^-$, $A_{1g}^- + B_{1g}^- + E_u^+$ density waves. In the $A_{1g}^- + B_{1g}^-$ state, $\mathcal{T}$ is not broken and we have gapless lines. In the $A_{1g}^- + B_{1g}^- + E_u^+$ state, the density wave order takes a form,

$$\Delta_{\mathbf{k}} = i\Delta_{A_{1g}^-}(\cos k_x + \cos k_y) + i\Delta_{B_{1g}^-}(\cos k_x - \cos k_y) + \Delta_{E_u^+} \sin k_x \cos k_y,$$

which has two straight vortex lines along the $k_z$ direction, given by ($k_x = \pi/2, k_y = \pm\pi/2$). These vortex lines cross the Fermi surface, and we have four Weyl nodes. Topological numbers for this state are $(2, 0)$.

For both cases, the transition from trivial $A_{1g}^+$ state to other density waves roughly occurs at $V_{\rm nnn}^{\rm h}/V_{\rm nn} = 0.35$, with $V_{\rm nnn}^z/V_{\rm nn}$ fixed at 0.2, which agrees with the estimate from Supplementary Equation 4.

## Supplementary References


[1] Weinberg, S. Effective action and renormalization group flow of anisotropic superconductors. *Nuclear Physics B* **413**, 567 – 578 (1994).

[2] Shankar, R. Renormalization-group approach to interacting fermions. *Rev. Mod. Phys.* **66**, 129–192 (1994).

[3] Raghu, S., Kivelson, S. A. & Scalapino, D. J. Superconductivity in the repulsive hubbard model: An asymptotically exact weak-coupling solution. *Phys. Rev. B* **81**, 224505 (2010).

[4] Kiesel, M. L. & Thomale, R. Sublattice interference in the kagome hubbard model. *Phys. Rev. B* **86**, 121105 (2012).

[5] Glaetzle, A. W. *et al.* Quantum Spin Ice and dimer models with Rydberg atoms. *Phys. Rev. X* **4** 041037 (2014).
